# Herbivory Protection via Volatile Organic Compounds Is Influenced by Maize Genotype, Not *Bacillus altitudinis*-Enriched Bacterial Communities

**DOI:** 10.3389/fmicb.2022.826635

**Published:** 2022-05-02

**Authors:** Sierra S. Raglin, Angela D. Kent, Esther N. Ngumbi

**Affiliations:** ^1^Microbial Ecology Laboratory, Department of Natural Resources and Environmental Sciences, University of Illinois, Urbana-Champaign, Urbana, IL, United States; ^2^Departments of Entomology, University of Illinois at Urbana-Champaign, Urbana, IL, United States

**Keywords:** herbivore-induced plant volatile (HIPV), plant-growth promoting bacteria (PGPB), volatile organic component (VOC), *Bacillus altitudinis*, maize (*Zea mays* L.)

## Abstract

Belowground, plants interact with beneficial soil microbes such as plant growth-promoting rhizobacteria (PGPR). PGPR are rhizosphere bacteria that colonize roots and elicit beneficial effects in plants such as improved plant growth, pathogen resistance, abiotic stress tolerance, and herbivore protection. Treatment of plants with PGPR has been shown to trigger the emission of volatile organic compounds (VOCs). Volatile emissions can also be triggered by herbivory, termed herbivore-induced plant volatiles (HIPV), with important ramifications for chemical-mediated plant and insect interactions. Much of our current understanding of PGPR and herbivore-induced volatiles is based on studies using one plant genotype, yet domestication and modern breeding has led to the development of diverse germplasm with altered phenotypes and chemistry. In this study, we investigated if volatile emissions triggered by PGPR colonization and herbivory varies by maize genotype and microbial community assemblages. Six maize genotypes representing three decades of crop breeding and two heterotic groups were used, with four microbiome treatments: live or sterilized soil, with or without a *Bacillus* inoculant. Soil sterilization was used to delay microbiome establishment, resulting in low-diversity treatments. At planting, maize seeds were inoculated with PGPR *Bacillus altitudinis* AP-283 and grown under greenhouse conditions. Four weeks post planting, plants were subjected to feeding by third instar *Helicoverpa zea* (Lepidoptera: Noctuidae) larvae. Volatiles were collected using solid phase microextraction and analyzed with gas chromatography-mass spectrometry. Illumina NovaSeq 16S rRNA amplicon sequencing was carried out to characterize the rhizosphere microbiome. Maize genotype significantly influenced total volatile emissions, and relative abundance of volatile classes. We did not document a strong influence of microbe treatment on plant VOC emissions. However, inoculating plants with PGPR improved plant growth under sterile conditions. Taken together, our results suggest that genotypic variation is the dominant driver in HIPV composition and individual HIPV abundances, and any bacterial-mediated benefit is genotype and HIPV-specific. Therefore, understanding the interplay of these factors is necessary to fully harness microbially-mediated benefits and improve agricultural sustainability.

## Introduction

Plants mount a medley of chemical defenses against insect herbivory, including indirect inducible defenses such as herbivore-induced volatile compounds (Kessler and Baldwin, 2001). Herbivore-induced plant volatile compounds (HIPV) act as chemical beacons to insect predators and parasitoids, allowing them to locate insect pests, promoting plant resilience to insect pests (War et al., 2011). Interest in HIPV utilization for insect pest management has increased in recent years, however, exploiting HIPVs for pest management requires a thorough understanding of the factors that can influence their production, composition, quality and quantity (Turlings and Erb, 2018). Recent evidence has pointed to the role of soil microbiota in HIPV production, depending on agricultural management (Malone et al., 2020). Agricultural manipulation of plant growth promoting rhizobacteria (PGPR), such as *Bacillus* species (Kloepper et al., 2004; Radhakrishnan et al., 2017), has been shown to elicit changes in plant immune response to biotic stressors, like pathogens and insects (Khan et al., 2020). As PGPR are projected to be a fundamental tool in future agricultural sustainability through conferring stress resilience without synthetic chemicals (Kenneth et al., 2019), it is important to assess conditions under which PGPR can promote herbivore resistance. *Bacillus* PGPR may interact with the plant immune system (Choudhary and Johri, 2009; Shafi et al., 2017) to mitigate herbivory, but more research is needed to explore PGPR influence on HIPV production, particularly in major agricultural crops, like maize. Identifying mechanisms of HIPV elicitation, such as the use of *Bacillus* PGPR, can support the sustainable resiliency of maize and other major field crops, reducing the adverse impacts of industrial pesticides and additives.

Numerous biochemical pathways are evoked upon insect herbivory. Leaf herbivory by chewing generalists elicits the production of various intracellular and extracellular metabolites (Ali and Agrawal, 2012). For instance, indole-derived benzoxazinoids are released intracellularly from vacuoles (Oikawa et al., 2001), and extracellularly within root exudates to modulate herbivore growth and recruit PGPR *Pseudomonas putida* to the root zone, assisting in plant defense (Neal et al., 2012). Additionally, herbivore-induced volatile compounds (HIPV) have been recognized as an indirect method of infochemical production (Maffei, 2010), recruiting parasitoids and predators of herbivores, as well as promoting conspecific priming of neighboring plants (Walling, 2000; Scala et al., 2013). Upon leaf damage, plants produce gaseous molecules, volatile organic compounds (VOCs) from numerous biochemical classes, including the oxylipin-derived jasmonates (Borrego and Kolomiets, 2016) and green-leaf volatiles (GLV), terpenoids (Arimura et al., 2009; Block et al., 2019), as well as aromatic amino acid derivates, like indole (Dudareva et al., 2004; D’Alessandro et al., 2006; Maffei, 2010). Ethylene, the first gaseous hormone discovered, is also essential in defense signaling cascades and elicitation of induced systemic resistance post-herbivory (Farag et al., 2013), solidifying the fundamental importance of volatile compounds in plant resiliency to biotic stress. HIPV blends vary by plant genotype (Gouinguené et al., 2001; Block et al., 2018). The blend itself is important for signaling, as well as changes in the abundance of specific HIPVs (Hoballah et al., 2002). As plant biochemical capacity is a function of genotype, as well as resources available for allocation and synthesis of defense metabolites, understanding plant and environmental drivers of HIPV production will reveal the bounds of their potential as novel biological control of insect pests.

Evidence suggests HIPV vary based on maize genotype (Degen et al., 2004). Landrace maize produces the sesquiterpenes (*E*)-β-caryophyllene and β-farnesene in response to oviposition by parasitoid *C. marginventris*, suggesting older varieties may respond to the presence of the parasitoid for HIPV production (Tamiru et al., 2011). Alternatively, American maize produces less (*E*)-β-caryophyllene during herbivory by *Spodoptera littorlis* and *Diabrotica virgifera vergifera* than teosinte (Köllner et al., 2008). (*E*)-β-caryophyllene attracted predators of both herbivores: leaf-chewing *Spodoptera* attracted parasitoid *C. marginventris* by producing (*E*)-β-caryophyllene in the leaves, while root pest *D. virgiferea* induced (*E*)-β-caryophyllene production only in the roots and recruited entomopathogenic nematode predators (Köllner et al., 2008). Interestingly, while the gene encoding *terpene synthase 23* (*tps23*), which produces (*E*)-β-caryophyllene, is maintained via positive selection in maize and teosinte, American maize varieties had significantly reduced transcription of *tp23*, when compared to teosinte and European maize (Köllner et al., 2008), indicating population divergence in HIPV through breeding practices. Moreover, treating maize with commercial resistance elicitors, BTH and Laminarin, reduced the production of numerous HIPV, including (*E*)-β-caryophyllene (Sobhy et al., 2012). However, this reduction was correlated with an increased attractiveness to numerous parasitoids, including *C. marginventris*, *Campoletis sonorensis*, and *Microplitis rufiventris* (Kokuiev) (Sobhy et al., 2012). Indole and aromatic HIPV production also vary significantly by genotype (Gouinguené et al., 2001). Indole has been shown to interfere with parasitoid attraction to herbivore-inflicted plants (D’Alessandro et al., 2006; Sobhy et al., 2012), and the mentioned commercial resistance elicitors reduced expression of maize indole synthase, implying a tradeoff between indole production and parasitoid attraction (Sobhy et al., 2012). Within teosinte, sesquiterpenes (*E*)-β-caryophyllene, (*E*)-β-farnesene, and (*E*)-bergamotene, were the most significant components of the HIPV blend in *Zea mays* subsp. *parviglumis* and *Zea diploperennis*, yet *Zea mays* subsp. *mexicana* had very low variation in HIPV blends (Gouinguené et al., 2001). These results suggest that genotypic variation is a strong driver of HIPV production, producing distinct inducible volatile profiles.

*Bacillus* is one of the most well-documented genera of plant-growth promoting rhizobacteria (PGPR) – root-zone inhabiting bacteria which positively influence plant health through various direct (nutrient acquisition, hormone production, etc.) and indirect (pathogen exclusion, antibiosis, etc.) mechanisms (Govindasamy et al., 2010; Bhattacharyya and Jha, 2012). *Bacillus* PGPR have been proposed as sustainable agricultural inoculants to assist in the maintenance of plant growth through one or more of these pathways (Radhakrishnan et al., 2017), making them an attractive solution for highly cultivated crops, like maize (*Zea mays*). However, a major drawback of using inoculants for herbivore resistance is insuring their persistence within the dynamic diversity of the native root microbiome. When combined with genotypic and environmental variation in the root microbiome and soil reservoir, as well as plant defense pathways, the beneficial effect of *Bacillus* on insect defenses may be lost. As insect herbivory results in massive yield losses in maize globally (Miedaner and Juroszek, 2021), research should focus on the efficacy of *Bacillus* in influencing resilience against herbivores. Therefore, a major goal for the utilization of *Bacillus* inoculants in maize agricultural systems is understanding their impact on maize herbivore defense signaling, and the role of the native microbiome and plant genotypic variation in mitigating *Bacillus* plant growth promoting activity.

*Bacillus* are gram-positive endospore forming bacteria known for their ability to withstand various environmental stressors, such as UV irradiation and desiccation, making them an attractive target for field-scale inoculation for plant growth promotion (Radhakrishnan et al., 2017). Numerous genera of *Bacillus* have been implicated in growth promotion. Plant-growth promoting *Bacillus* inoculants have been reported to confer abiotic stress tolerance, *e.g.*, resistance to salinity, drought, and heavy metals, to various crops species, including maize (*Zea mays*) and wheat (*Triticum aestivum*) (Sun et al., 2017; de Lima et al., 2019; Elfira et al., 2020; Yue et al., 2021). In addition to abiotic stress, *Bacillus* PGPR species can act as biocontrol agents against biotic stress from pathogens and pests (Yan et al., 2002; Lu et al., 2017). *Bacillus* can directly act as biocontrol agents by producing hydrolytic enzymes or antimicrobial agents, suppressing pathogen growth and survival (Hashem et al., 2019). Antagonistic effects against biotic stressors have been reported with numerous *Bacillus* species, such as *Bacillus subtilis*, *B. amyloliquefaciens*, and *B. altitudinis* (Chowdhury et al., 2015; Sunar et al., 2015; Shafi et al., 2017). Indirect methods of biocontrol occur through the production of various plant hormones, like indole-acetic acid, ethylene, and salicylic acid (Patten and Glick, 1996; Ali et al., 2015). Additionally, *Bacillus* species have been recorded modifying the activity of metabolic pathways involved in defense metabolite synthesis. Endophytic *Bacillus altitudinis* modifies transcriptional expression of numerous defense-related genes, like phenylalanine ammonia lyase and phenol oxidase (Hasan et al., 2020). Recent research has also suggested the influence of bacterial volatile compounds (BVCs) in plant response to biotic stressors, the effects of which are due to various mechanisms like direct inhibition of insect or pathogen growth, or modification of plant defense pathways (Cellini et al., 2021). The stimulation of defense-related gene expression by plant growth promoting bacteria is referred to induced systemic resistance (ISR; Kloepper et al., 2004). Interactions between *Bacillus* inoculants and plant immune pathways prime plant defenses for rapid response to herbivore damage.

Modifying microbiomes for plant growth and yield is an attractive solution for maximizing agricultural sustainability but the efficacy of microbial inoculants is under thorough investigation. This is particularly important for complex tri-trophic ecological interactions, such as parasitoid/predator recruitment through HIPV production. Evidence suggests that agricultural management that alters plant-soil feedbacks, such as companion cropping or cover cropping, can alter production or composition of plant defense compounds and herbivore response (Mutyambai et al., 2019; Wang et al., 2019). Pangesti et al. (2015) recorded the release of (E)-α -bergomatene during *Pseudomonas fluorescens* colonization of *Arabidopsis thaliana* roots, which increased recruitment of parasitic wasp *Microplitis mediator* to herbivore-infested leaves (Pangesti et al., 2015). However, the specific contributions of plant-growth promoting *Bacillus* to indirect inducible defense signaling are unknown. *Bacillus altitudinis* has be recorded displaying plant beneficial activity through growth promotion (seed yield and phenology) in maize (Elfira et al., 2020; Zhang et al., 2021). Zhang et al. (2021) reported maize inoculated with *B. altitudinis* showed increased fresh weight (g) and size (Zhang et al., 2021). In *Miscanthus x giganteus* and wheat (*Triticum aestivum*), *B. altitudinis* maintained biomass yield during growth in metal contaminated soils (Pranaw et al., 2020; Yue et al., 2021). *B. altitudinis* application upregulated stress tolerance genes in numerous plant species, like phenylpropanoid and lipoxygenase synthesis genes (Yue et al., 2021; Zhang et al., 2021), and produces indole acetic acid (Sun et al., 2017), indicating it has the capacity to modulate biochemical pathways important for inducible defense metabolite production. However, it is currently unknown if *B. altitudinis* has the capacity to modify herbivore-induced plant volatiles (HIPV), and if its preferred functions are observable within the rhizosphere microbiome. Moreover, as evidence suggests maize genotype is a strong driver of HIPV composition (Gouinguené et al., 2001), it is important to assess the beneficial functions of PGPR across genotypes. Therefore, the purpose of this research is to understand how the rhizosphere microbiome and maize genotype influence the abundance and composition of HIPV, and if the addition of *B. altitudinis* distinctly modifies the induced volatilome.

## Materials and Methods

### Seed Selection and Preparation

Maize (*Zea mays* L. subsp. *mays*) seeds were obtained from the USDA North Central Region Plant Introduction Station (Ames, Iowa). Six inbred maize genotypes (Table 1), within two heterotic groups (Stiff Stalk and Non-Stiff Stalk), were chosen for this study. Genotypes were chosen to represent eras which span US pesticide usage during the Green Revolution.

**TABLE 1:** Maize genotypes used during this study.

Genotype	Heterotic Group	Decade
B14*	Iowa Stiff Stalk	1950s
B73	Iowa Stiff Stalk	1970s
PHJ40	Iowa Stiff Stalk	1980s
OH43	Non-Stiff Stalk	1940s
Pa91	Non-Stiff Stalk	1970s
PHG84	Non-Stiff Stalk	1980s

*Asterisk denotes genotypes which had insufficient germination and were removed from all analyses.*

Seeds were rinsed with sterilized deionized (DI) H_2_O, washed briefly with 70% ethanol, soaked in 1.5% NaClO solution for 5 minutes, followed by 5 additional DI H_2_O rinses. Sterilized seeds were germinated on sterile petri dishes, lined with autoclaved filter paper, within incubation chambers set at 25°C, without supplemental lighting.

### Plant Growth Conditions

Seeds were sown in 16.51 cm Azalea pots with steam-sterilized Berger BM7 45% soilless mix (Composted pine bark, coarse peat moss, parboiled rice hulls, dolomitic and calcitic limestone) and grown for 30 days. Three seeds were planted per pot, and thinned to one plant per pot after emergence. The soilless mix was inoculated with a 10% field soil, which was air-dried and sieved (2 mm), prior to incorporation with the planting medium. Field soil was collected from a secondary successional field, to avoid agricultural legacy effects on the microbial community. Soil treatments (sterilization and inoculation) are described below. Plants were grown at 25°C, with 16L:8D photoperiod, and watered via an automatic irrigation system, as needed. Fertilizer was applied as 20-20-20 NPK plus micronutrients fertilizer weekly. The pots were set up in a randomized block design within the greenhouse, with block as a random effect, and transported to an entomology greenhouse for herbivore application and HIPV collection prior to destructive soil sampling.

### Plant Growth-Promoting Rhizobacteria Selection and Application

*Bacillus altitudinis* strain AP-283 was obtained from Auburn University. AP-283 was chosen from a collection of *Bacillus* PGPR selected based on their capacity to promote growth of maize (Calvo et al., 2017), and to induce emission of VOCs in cotton and maize (Kloepper et al., 2013) and confer herbivory protection in maize (Disi et al., 2018a,b).

*Bacillus altitudinis* strain AP-283 was grown on tryptic soy agar (TSA) plates and diluted in sterile distilled water to achieve a final OD_600_ of 1.0. Each planted seed received aliquots of 1mL AP 283 solution at planting. Following thinning, each plant received 1mL weekly for 4 weeks, to ensure artificial persistence of AP-283.

To explore the synergistic role of the soil microbiome and *Bacillus altitudinis* inoculant in herbivory resistance, four microbial treatments (n = 5) were applied to each maize genotype: *Bacillus* with sterilized soil (BS), *Bacillus* with 10% live microbiome inoculum (BL), live microbiome inoculum without *Bacillus* (L), and sterilized soil without *Bacillus* or live soil inoculum (S). In addition to steam sterilization, the Berger BM7 soil mixture + 10% field soil inoculum (BS and S treatments) was sterilized three times for one hour, each, in the autoclave, for a total of 3h. Sterilization was used to significantly reduce rhizosphere microbial diversity and delay rhizosphere microbiome establishment, as maintaining sterilization in a greenhouse setting is non-plausible.

### Herbivory Treatment

Corn earworm (*Heliocoverpa zea*) was selected for the induction of herbivore-induced volatile compounds (HIPV). Third instar larvae were purchased from Benzone Research (Carlisle, PA, United States). Insects were starved for 6 h before feeding on corn plants to induce the emission of volatiles. Four larvae per plant were used. Larvae were gently transferred onto maize plants whorls. Larvae fed for 24 h prior to volatile collection.

### Herbivore-Induced Volatile Compound Collection and Analysis

Solid phase micro extraction (SPME) fiber coated with poly dimethyl siloxane-divinyl benzene (PDMS/DVB, 65 μm) purchased from Supelco (Bellefonte, PA, United States) was used to collect herbivore-induced volatile organic compounds following protocols similar to those reported by Ngumbi and Ugarte (2021). One hour prior to headspace volatile collection, maize plants were wrapped with an odor-blocking oven plastic bag (Arcadia INTL, EL Monte, CA), to allow for volatile concentration. One hour later, the SPME was introduced into the sealed plant through an insertion space created with thin forceps. The SPME fiber was exposed and the headspace volatiles (VOCs) were collected for 40 minutes. Immediately after VOCs collection, the fiber was injected into a Hewlett-Packard (HP) 6890 GC (Hewlett-Packard, Sunnyvale, CA, United States) in splitless mode, interfaced to an HP 5973 mass-selective detector (MSD), with helium carrier gas. The column was programmed from 40°C/2 min, with increases by 5°C/min to 200°C. Injector and transfer line temperatures were set at 200°C. Identification of peaks was done by using NIST 98 library (National Institute of Standards and Technology, Gaithersburg, Maryland) and by comparing with published GC profiles of maize head space volatiles (Thompson et al., 1971; Loughrin et al., 1994; McCall et al., 1994; Gouinguené et al., 2001; Pinto-Zevallos et al., 2016; Ngumbi and Ugarte, 2021). The structures of the identified volatile compounds were confirmed using commercially available synthetic standards with purity > 97% (as indicated on the labels) obtained from Sigma^®^ Chemical Co. (St. Louis, Missouri).

### Soil and Plant Sampling

After thirty days of growth, maize plants were destructively sampled by removing the entire root zone from their corresponding pots. Aboveground biomass was removed, weighed, and oven dried. Root systems were stored at 4°C until cleaning, followed by thorough rinsing and oven drying. Root:shoot ratio was quantified using dry root biomass and dry aboveground biomass. Soil adhering to the roots, hereafter rhizosphere soil, was collected in bags and briefly stored at 4°C until processed. The rhizosphere soil was then mechanically mixed, and put into 15 mL centrifuge for lyophilization, then frozen at −20°C while awaiting DNA extractions.

### DNA Extractions

Fifty milligrams of freeze-dried soil were weighed into microcentrifuge tubes for DNA extractions on the QiaCUBE HT DNA extraction robot (Qiagen, Germany), using the Qiagen DNAEasy soil extraction kit and protocol. The Qiagen DNAEasy soil kit does not require DNA cleaning with CTAB. Immediately prior extractions, DNA was frozen at −20 °C while awaiting Qubit and NanoDrop quantification. Genomic DNA was submitted to the Roy J. Carver Biotechnology Center at the University of Illinois Urbana-Champaign (Urbana, IL) for 16S rRNA Amplicon Sequencing.

### 16S rRNA Amplicon Sequencing and Sequence Processing

Illumina NovaSeq was used for 16S rRNA amplicon sequencing, conducted at the Roy J. Carver Biotechnology Center (Urbana, IL) to assess the contributions of microbe treatments on HIPV relative abundance and composition. 16S rRNA gene amplicon sequencing used the 515F (5′-GTGYCAGCMGCCGCGGTAA-3′) and 806R (5′-GGACTACVSGGGTATCTAAT-3′) primers to amplify the V4 region of the prokaryotic 16S rRNA gene (Caporaso et al., 2011). The DNA sequencing and bioinformatics workflow has been previously detailed in Favela et al. (2021). Briefly, forward and reverse paired-end sequences were merged using Fast Length Adjustment of Short reads (FLASH) software (Magoč and Salzberg, 2011). Once merged, reads were filtered using a minimum quality score of 30 for 90% of removed bases via the FASTX-Toolkit (Gordon and Hannon, 2010). FASTQ reads were then converted to FASTA format. Sequences were binned based on 97% similarity into operational taxonomic units (OTUs) using USEARCH version 8.1.1861 (Edgar, 2010). Quantitative Insights into Microbial Ecology (QIIME) was used solely used to generate an OTU table and to assign taxonomy for downstream analyses in R (Kuczynski et al., 2011). After OTU taxonomic assignment, mitochondrial and chloroplast OTUs were removed. The OTU table was rarefied and singleton OTUs were removed prior to statistical analyses. One outlier sample was removed due to low read counts. Results from amplicon sequencing are reported in the supplemental information. Amplicon sequence data for 16S rRNA genes is available for download on the NCBI SRA database at BioProject accession number PRJNA785015.

### Statistical Analyses

All statistical analyses were performed in R Studio statistical software (Version 4.1.2, 2021) (R Core Team, 2021; Venables et al., 2021). All figures were produced using *ggplot2* v. 3.3.5 (Wickham, 2016). The influence of genotype and microbiome treatment (BS, S, L, BL) on plant growth parameters (root dry biomass, above ground dry and wet biomass, root:shoot), were assessed using two-way mixed effects model, with block as a random effect using the *lme4* package v. 1.1-27 (Bates et al., 2015). B14, a stiff stalk genotype used in this study, had incomplete germination and was excluded from HIPV analyses. Due to this, there is an unbalanced design when comparing heterotic groups, with the Non-Stiff Stalk containing three genotypes, while the Stiff Stalk heterotic group contains only two genotypes. The influence of heterotic group was, therefore, ignored for the remainder of the analyses. Conditional (variance explained by the full model) and marginal (variance explained by fixed effects) coefficients of determination (R^2^_c_, and R^2^_m_, respectively) were calculated using the r.squaredGLMM() function of the *MuMIn* package v. 1.43.17 (Barton, 2020).

**FIGURE 1:**
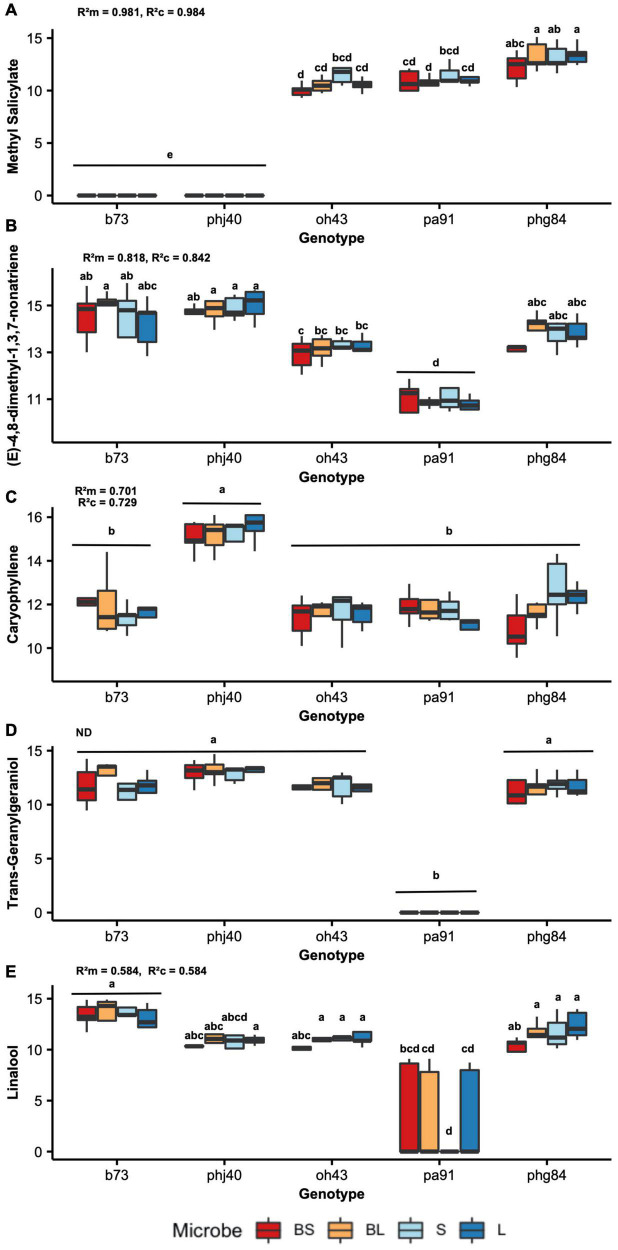
Individual herbivore-induced volatile compound (HIPV) quantities by maize genotype (*x*-axis) and microbe treatment (color, see key on bottom). Random forest model was used to rank individual HIPV based on importance (using MDA score). Peak area quantities were log10 + 1 transformed to assume normality. Tukey’s honestly significant difference was used to group means, the results of which are denoted on each boxplot. 2-Way ANOVA coefficients of determination (conditional and marginal, R^2^_c_, and R^2^_m_, respectively) are indicated on each graph: **(A)** Methyl salicylate, **(B)** (E)-4,8-dimethyl-1,3,7-nonatriene, **(C)** caryophyllene, **(D)** Trans-Geranylgeraniol, and **(E)** linalool are shown. Coefficient of determination of Trans-Geranylgeraniol is not shown, due to violations of assumptions (denotes with “ns”).

The interaction of genotype and microbiome treatment on the relative abundance of HIPV compounds classes, as well as individual HIPV compounds, was also assessed using a mixed effects ANOVA. The major compound classes were as follows: sesquiterpenes, monoterpenes, green leaf volatiles, esters, ketones, alkane hydrocarbons, diterpene alcohols, benzoate esters, and other. A Random Forests model using package *randomForest* v 4.6-14 was used to rank individual volatile compounds by importance (Ngumbi and Ugarte, 2021). The top ten compounds are shown in Table 2 with Mean Decrease Accuracy (MDA) scores, and the top five are visualized in Figure 1. Two-way mixed effects ANOVAs were then used to assess influence of genotype and microbe on top HIPV compounds. Shapiro-Wilk test was used to identify deviations in residual variances of all models and calculated with the shapiro test function of the *stats* package v. 4.1.1.; W > 0.9 was used to indicate normally distributed residual variances. Levene’s Test for homogeneity of variances across microbiome and genotype groups was calculated using Levene Test function of the *car* package v. 3.0-11. Type III analysis of variance table with Satterthwaite’s method is reported in Tables 1, 3 by using the anova function of the *stats* package v. 4.1.1 (R Core Team, 2021). Data was log_10_ transformed to assume normality, where required. Means were separated using Tukey’s honestly significant difference test using the HSD.test function from the *agricolae* package v. 1.3-5 (De Mendiburu, 2021), for plant parameters and total HIPV models.

**TABLE 2:** Ranking of Importance of 36 identified herbivore-induced volatile compounds (HIPV) based on maize genotype and rhizosphere microbiome treatment.

Ranking	HIPV	MDA
1	Methyl salicylate	39.084279
2	(E)-4,8-dimethyl-1,3,7-nonatriene*	26.724274
3	Caryophyllene	24.240676
4	trans-Geranylgeraniol	23.811890
5	Linalool	22.880034
6	β-Myrcene	22.070551
7	trans-α-Bergamotene	19.432684
8	epi-Bicyclosesquiphellandrene	16.791102
9	Germacrene D	16.057696
10	D-Limonene	15.561633

A Random Forest decision tree was used and rankings were determined using Mean Decrease Accuracy (MDA). *Previously identified as (E)-2-Butenoic acid, 2-(methylenecyclopropyl)prop-2-yl ester (Xavier et al., 2011).

**TABLE 3:** Type III analysis of variance table with Satterthwaite’s method of treatment (Genotype, Microbe) on plant growth parameters (root biomass, dry aboveground biomass, wet aboveground biomass, and root:shoot ratio).

Fixed Effects	SS	MS	DF	*F*-Value	R^2^_m_	R^2^_c_
**Root: Shoot**					0.4859034	0.5211005
Microbe***	2.9203	0.97343	3	17.3425		
Genotype***	1.3154	0.32885	4	5.8588		
M x G*	1.5532	0.12943	12	2.3060		
**Dry Shoot Biomass (g DW)**					0.231221	0.3526322
Microbe**	19.736	6.5787	3	4.8403		
Genotype*	16.007	4.0018	4	2.9444		
M x G	11.872	0.9894	12	0.7279		
**Wet Shoot Biomass (g)**					0.2900259	0.4952666
Microbe**	1766.28	441.57	3	6.6632		
Genotype***	1025.07	341.69	4	5.1560		
M x G	981.68	81.81	12	1.2344		
**Root Biomass (g DW)**					0.6328723	0.6328723
Microbe***	208.14	69.379	3	26.2236		
Genotype***	123.26	30.814	4	11.6471		
M x G ***	114.21	9.517	12	3.5973		

Dependent variable is bolded above model fixed effects. Asterisk denote factor significant: P = 0 ‘***’; 0.001 ‘**’; 0.01 ‘*’; 0.05 ‘.’; Marginal (Fixed Effects) and Conditional (Full Model) coefficients of determination for each model are reported.

The composition of HIPV compounds was assessed using a non-metric dimensional analysis (NMDS) on the Bray-Curtis’s dissimilarity matrix created from the volatiles profile (Ngumbi and Ugarte, 2021) to understand how the overall blend of HIPV changes with genotype and microbiome treatment, using the metaMDS function of the *vegan* package v. 2.5-7 (Oksanen et al., 2020). PERMANOVA was conducted using the adonis function in *vegan* to identify significant contributions of genotype and microbiome treatment on HIPV blend. The contributions of plant data (root, dry and wet aboveground biomass, and root:shoot ratio), as well as microbial richness (observed, chao1, Shannon diversity index) to HIPV composition was assessed using the envfit function within the *vegan* package v. 2.5-7.

To better understand the influence of the microbiome on HIPV, a Mantel test was used to identify Spearman correlations between the 16S rRNA dissimilarity and HIPV dissimilarity matrices, using Bray-Curtis distances. The bacterial 16S rRNA OTU table was Hellinger transformed, prior to the construction of a Bray-Curtis dissimilarity matrix, and the Mantel test was conducted using the *vegan* package v. 2.5-7 in R, with 9999 permutations.

## Results

### Treatment Influence on Plant Growth Parameters

**FIGURE 2:**
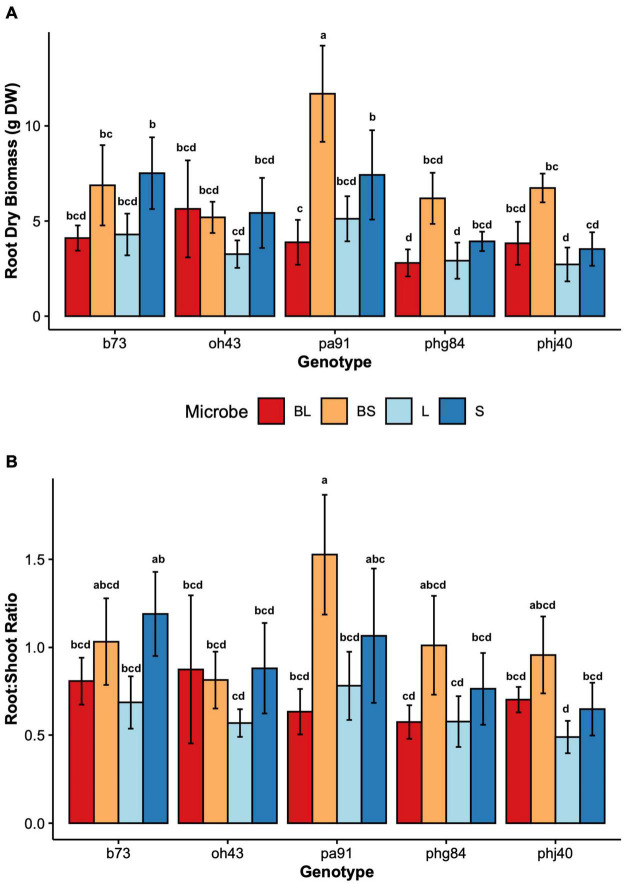
Root growth parameters by maize Genotype (B73, PHJ40, Pa91, OH43, PHG84) and Microbe treatment: Bacillus+Live soil (BL), Bacillus+Sterile soil (BS), Sterile soil (S), Live soil (L). **(A)** Dry root biomass in grams dry weight after washing and oven drying; **(B)** ratio of below to aboveground biomass, root:shoot ratio. Bars represent mean ± 2 SEM. Mean separation groups from Tukey’s honestly significant difference test placed above SEM bars.

**FIGURE 3:**
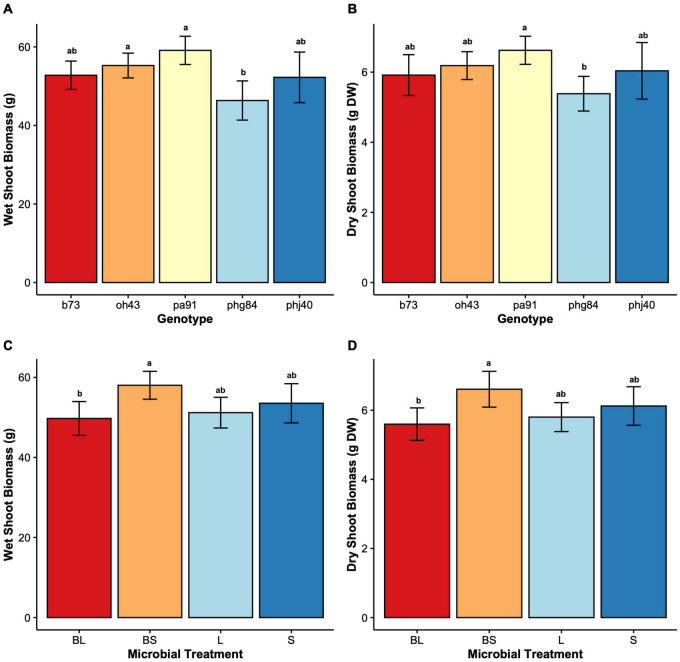
Shoot growth parameters by Genotype (B73, PHJ40, Pa91, OH43, PHG84) and Microbe treatment: Bacillus+Live soil (BL), Bacillus+Sterile soil (BS), Sterile soil (S), Live soil (L). **(A)** wet shoot biomass in g wet weight, displayed by Genotype; **(B)** dry shoot biomass in grams dry weight after oven drying, displayed by Genotype; **(C)** wet shoot biomass in g wet weight, displayed by Microbe treatment; **(D)** dry shoot biomass in g wet weight, displayed by Microbe. Bars represent mean ± 2 SEM. Genotype x Microbe group means were separated using Tukey’s honestly significant difference test placed, the results of which are placed above SEM bars.

Results of two-way mixed effects models are reported in Tables 3, 4. Microbiome treatment (BS, BL, L, S) and genotype impacted plant growth. Dry root biomass and root:shoot (R:S) ratio was significantly influenced by the interaction between genotype and microbiome (Figure 2), indicating the genotypic influence on root investment varies based on the available soil microbiota (Table 3). Microbe (*P* < < 0.01) treatment and genotype (*P* < 0.02) impacted aboveground investment (wet and dry biomass), as well, but independently (Figure 3).

**TABLE 4:** Type III analysis of variance table with Satterthwaite’s method of treatment (Genotype, Microbe) on HIPV compound classes and total HIPV.

Fixed Effects	SS	MS	DF	*F*-Value	R^2^_m_	R^2^_c_
**Total HIPV**					0.5355116	0.5576677
Microbe**⋅**	1.0988e+14	3.6626e+13	3	2.5823		
Genotype***	1.4381e+15	3.5953e+14	4	25.3481		
M x G	1.6484e+14	1.3737e+13	12	0.9685		
**Sesquiterpene**					0.4697384	0.5417335
Microbe	0.00992	0.00331	3	0.1655		
Genotype***	1.82507	0.45627	4	22.8503		
M x G	0.15548	0.01296	12	0.6489		
**Ketone**					0.4066252	0.4661853
Microbe	0.01257	0.004188	3	0.2905		
Genotype***	0.93408	0.233521	4	16.1971		
M x G	0.12900	0.010750	12	0.7456		
**Alkane Hydrocarbon**					–0.3468411	–0.4022934
Microbe	0.0002141	0.00007137	3	0.8857		
Genotype***	0.0038702	0.00096755	4	12.0080		
M x G	0.0004780	0.00003983	12	0.4944		
**Monoterpene**					0.4440484	0.4837898
Microbe	0.00532	0.001773	3	0.1914		
Genotype ***	0.73526	0.183815	4	19.8433		
M x G	0.03615	0.003013	12	0.3252		
**Green Leaf Volatile**					0.3861213	0.4044357
Microbe	0.0011729	0.00039098	3	1.4911		
Genotype***	0.0106452	0.00266130	4	10.1494		
M x G	0.0047923	0.00039936	12	1.5230		
**Ester**					0.535072	0.6271043
Microbe	0.00211	0.000704	3	0.0908		
Genotype***	1.06662	0.266655	4	34.4277		
M x G	0.02088	0.001740	12	0.2246		
**Benzoate Ester**°						
**Diterpene Alcohol** ^§^					0.2894044	0.4427281
Microbe	0.0002044	0.00006815	3	0.6577		
Genotype***	0.0047449	0.00118624	4	11.4494		
G x M	0.0003418	0.00002849	12	0.2749		
**Other**					0.3437729	0.41034
Genotype***	0.0043783	0.0010946	4	11.6835		
Microbe	0.0001560	0.0000520	3	0.5550		
G x M	0.0007644	0.0000637	12	0.6799		

Dependent variable is bolded above model fixed effects. Asterisk denote factor significant: P = 0 ‘***’; 0.001 ‘**’; 0.01 ‘*’; 0.05 ‘**⋅**’; Marginal (Fixed Effects) and Conditional (Full Model) coefficients of determination for each model are reported. ^§^ = dependent variable was log10 transformed to assume normality. ° = denotes models which violated assumptions after transformations.

### Herbivore-Induced Volatile Compounds

A total of 36 volatile compounds were detected following by corn ear worm (*Heliocoverpa zea*) feeding on five maize genotypes (B73, PHJ40, OH43, PHG84, PA91) under four microbiome treatments: live soil (L), sterile soil (S), *Bacillus altitudinis* AP-283 applied to sterile soil (BS), and *B. altitudinis* AP-283 applied to live soil (BL). The detected HIPV were classified as ketone (cyclopentanone, 2-ethylcyclopentanone, [1,1′-bicyclopentyl]-2-one), monoterpenes (β-myrcene, D-limonene, trans-β-ocimene, γ-terpinene, linalool), sesquiterpenes (α-cubebene, α-muurolene, ylangene, α-copaene, β-bourbonene, α-sesquiphellandrene, cis-muurola-3,5-diene, caryophyllene, epi-bicyclosesquiphellandrene, trans-α-bergamotene, humulene, (*E*)-β-farnesene, β-copaene, γ-muurolene, germacrene D, β-guaiene, trans -γ-cadinene, σ-cadinene), alkane hydrocarbons (heptadecane, pentadecane), diterpene alcohol (trans-geranylgeraniol), benzoate ester (methyl salicylate), ester ((*E*)-4,8-dimethyl-1,3,7-nonatriene)), green leaf volatile (3-hexen-1-ol,acetate (*Z*)), and other (bicyclopentyl-1,1′-diene, 4-cyanocyclohexene).

### Treatment Influence on Herbivore-Induced Volatile Compounds

**FIGURE 4:**
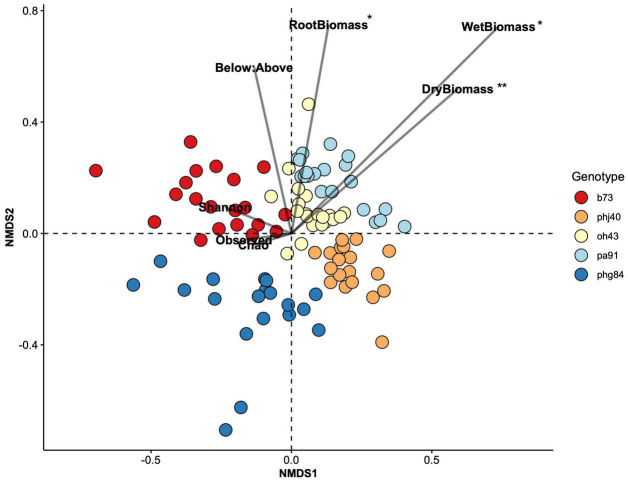
Non-metric multidimensional scaling (NMDS) ordination constructed with a Bray-Curtis dissimilarity matrix of HIPV peak area data. Plant biomass (Shoot dry and weight biomass, dry root biomass, root:shoot ratio) and rhizosphere bacterial community richness parameters (observed richness, Chao1 index, Shannon Evenness) were correlated to NMDS1 and NMDS2 points using the envfit() function of the *vegan* package in R; significance is reported in Table 7. Significant vectors displayed with an asterisk: *P* = 0 ‘***’; 0.001 ‘**’; 0.01 ‘*’; 0.05 ‘.’.

Non-metric multidimensional scaling analysis of the HIPV Bray-Curtis dissimilarity matrix revealed strong clustering due to genotype (Figure 4). PERMANOVA analyses confirmed that genotype strongly influenced NMDS structure (Table 5, R^2^ = 0.47466, *P* = 0.001). Microbe treatment, via manipulation of the microbial community within the potting mix via sterilization (sterile vs. live) and the addition of *Bacillus altitudinis* strain AP-283, did not significantly influence HIPV composition, determined via PERMANOVA, and did not significantly interact with genotype (Supplementary Figures 1-3). This was further supported with non-significant results of the Mantel test (*r* = −0.05, *P* = 0.965), indicating no correlation between HIPV and bacterial 16S rRNA Bray-Curtis distance matrices.

**TABLE 5:** Permutational multivariate analysis of variance (PERMANOVA) table.

Factors	Degrees of Freedom	Sums of Squares	Mean Squares	*F*-Value	R^2^
Microbe	3	0.3195	0.10650	0.9145	0.01599
Genotype***	4	9.4848	2.37121	20.3629	0.47466
M x G	12	0.8621	0.07184	0.6170	0.04314
Residuals	80	9.3158	0.11645		0.46620
Total	99	19.9822			1.00

Herbivore-induced plant volatiles (HIPV) Bray’s Curtis Dissimilarity matrix was subjected to PERMANOVA to identify significant influence of Microbe treatment (BS, BL, L, S) and Genotype (B73, PHJ40, PHG84, OH43, PA91) on HIPV composition. Bray’s dissimilarity matrix was produced using vegdist() function and the PERMANOVA was conducted using the adonis() function, both of the vegan package. Block was used as the constraining strata condition. Asterisk denote factor significance: P = 0 ‘***’; 0.001 ‘**’; 0.01 ‘*’; 0.05 ‘**⋅**’.

**TABLE 6:** Two-way analysis of variance table.

Volatile	Genotype			Microbe			Genotype x Microbe		
	df	F	P	df	F	P	df	F	P
A - Methyl Salicylate*	4,76	1600.8900	< 0.001	3, 76	3.0686	0.03287	12, 76	1.3638	0.20213
B - (E)-4,8-dimethyl-1,3,7-nonatriene*	4,76	125.7786	< 0.001	3, 76	1.2386	0.3016	12, 76	0.7606	0.6881
C - Caryophyllene*	4,76	59.5968	< 0.001	3, 76	0.5181	0.6711	12, 76	1.3894	0.1896
D - Trans-Geranylgeraniol*	4,76	−	−	3, 76	−	−	12, 76	−	−
E - Linalool*	4,76	32.8971	< 0.001	3, 76	0.0671	0.9772	12, 76	0.6448	0.7977
F - β-Myrcene*	4,76	−	−	3, 76	−	−	12, 76	−	−
G -trans-α-Bergamotene	4,76	−	−	3, 76	−	−	12, 76	−	−
H - epi-Bicyclosesquiphellandrene*	4,76	48.6385	< 0.001	3, 76	0.7959	0.4999	12, 76	1.5576	0.1227
I - Germacrene D*	4,76	19.7076	< 0.001	3, 76	0.0849	0.9681	12, 76	0.4616	0.9309
J - D-Limonene*	4,76	12.2691	< 0.001	3, 76	0.5719	0.6353	12, 76	0.2722	0.9920

The top 10 important HIPVs, determined using MDA score via Random Forest Model, were subjected to two-way ANOVA’s with Block as a random effect. The HIPV peak area values were log_10_ + 1 transformed to assume normality. *Denotes volatiles which were log_10_ + 1 transformed. ‘−’ in column indicated models which violated ANOVA assumptions after outlier removal and data transformation

**TABLE 7:** Significant continuous variables influencing NMDS ordination structure.

Factors	NMDS1	NMDS2	R	P
Root Biomass*	0.1739470	0.9847550	0.09234417	0.011
Wet Shoot Biomass*	0.7045319	0.7096724	0.17473196	0.011
Dry Shoot Biomass**	0.7519955	0.6591682	0.09893165	0.001
Root: Shoot	−0.2171252	0.9761438	0.059398598	0.060
Observed Richness	−0.9910300	−0.1336396	0.004609559	0.810
Chao1 Richness	−0.9569455	−0.2902677	0.003172513	0.860
Shannon Evenness	−0.9281458	0.3722167	0.010365568	0.594

Plant growth parameters and microbial richness values were fit onto NMDS ordination to identify variables correlating to HIPV composition, using the envfit() function from the vegan package was used and the summary output is reported below. Asterisk denote factor significance: P = 0 ‘***’; 0.001 ‘**’; 0.01 ‘*’; 0.05 ‘**⋅**’.

The envfit function in the *vegan* package was used to identify continuous variables that were correlated to NMDS structure (Figure 4). Root dry biomass (R = 0.09, *P* = 0.011), shoot wet biomass (R = 0.175, *P* = 0.001), and shoot dry biomass (R = 0.099, *P* = 0.011) significantly influenced NMDS structure. Microbial community richness (Chao1, and Observed) and evenness (Shannon) did not significantly drive NMDS structure. Root:shoot ratio nearly correlated to NMDS structure (R = 0.059, *P* = 0.060).

**FIGURE 5:**
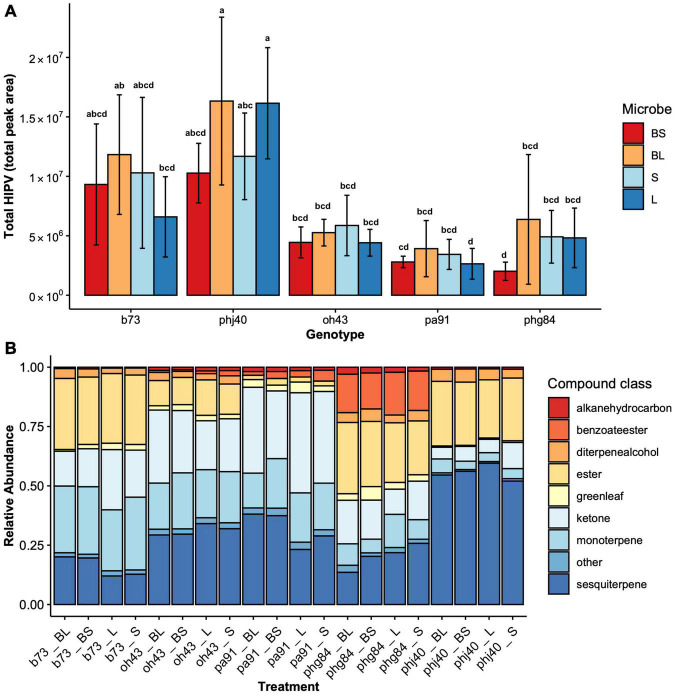
**(A)** Total HIPV (total peak area) across Genotype (B73, PHJ40, Pa91, OH43, PHG84) and Microbe treatment: Bacillus+Live soil (BL), Bacillus+Sterile soil (BS), Sterile soil (S), Live soil (L). The total peak area for each 36 identified HIPV was summed for each sample. Bars represent mean ± 2 SEM. Mean separation groups from Tukey’s honestly significant difference test placed above SEM bars. **(B)** Relative abundance of HIPV compound classes across Genotype and Microbe treatment; they are combined and represented as “Treatment.”

The total HIPVs produced (Figure 5A), and relative abundance of HIPV (Figure 5B) compound classes were significantly influenced by maize genotype (Table 4). Microbe treatment was significant in influencing total HIPV production (F = 2.5823, *P* = 0.05). Model summary identified a significant influence of the PHJ40 genotype in live soil (*P* = 0.01065) on total HIPV produced. All compound classes (monoterpenes, sesquiterpenes, esters, benzoate esters, ketones, green leaf volatiles, diterpene alcohols, alkane hydrocarbons, and other) were significantly influenced by genotypes. Relative abundances were not influenced by microbial treatment (Table 4). Benzoate Ester model was inconclusive due to violations of assumptions, even after data transformations.

Results of Random Forest model ranking of importance are reported in Table 2. Methyl salicylate was ranked as number one, based on MDA scores. Two-way mixed effects ANOVA results are reported in Table 6, for the top 10 ranked individual HIPV. Unfortunately, due to violations of ANOVA assumptions, trans-geranylgeraniol, β-myrcene, trans-α-bergamotene model results are not reported. Plant genotype significantly influenced all ranked individual volatiles (*P* < 0.001, Table 6). Only methyl salicylate was weakly influenced by microbe treatment (*P* = 0.03). Interestingly, methyl salicylate was not detected from B73 or PHJ40 genotypes in response to *Heliocoverpa zea* feeding, the two stiff-stalk varieties. Due to the lack of B14 germination, however, we are unable to determine if the lack of methyl salicylate production is specific to heterotic groups.

## Discussion

This study sought to understand how herbivore-induced plant volatile (HIPV) compounds are modulated by maize genotype and rhizosphere microbiota and if *Bacillus altitudinis* possessed the ability to modify production and composition of HIPV. Results suggest that genotypic variation in HIPV composition overshadowed *Bacillus* or microbiome-mediated HIPV release. Prior research has also recorded maize genotype influencing HIPV production (Gouinguené et al., 2001; Degen et al., 2004). For example, Degen et al. (2004) calculated the broad-sense heritability of total volatile emissions from 31 American and European inbred maize as H^2^ = 0.84. Specific volatile classes, like terpenes, may have even been maintained throughout maize breeding via stabilizing selection, indicating certain volatile compounds are functionally important for maize and its progenitors across environments (Köllner et al., 2009). This suggests that maize has maintain an impressive degree of HIPV genetic variability, and is a major control point for HIPV total emissions and blend. However, in our study, the Stiff-Stalk inbred variety PHJ40 did influence total volatile emissions within live soil treatments (Figure 5A). Recent evidence also supports a moderate genotypic influence on the diversity of the rhizosphere microbiome (Favela et al., 2021), which is also a heritable phenotype (Walters et al., 2018). This suggests that genotype-specific rhizosphere interactions influence HIPV load. As maize breeding with the goal of controlling insect pest has dominated breeding efforts, like Bt-maize (Horikoshi et al., 2021), the results of this study contribute to the growing body of evidence that HIPV could be genetically manipulated for maize germplasm development (Miedaner and Juroszek, 2021).

Thirty-six individual HIPV were induced upon herbivory by *Heliocoverpa zea*. Methyl salicylate (MeSA) was strongly influenced by maize genotype. Most interestingly, methyl salicylate was not detected in the stiff-stalk varieties, B73 and PHJ40, and was weakly influenced by Microbe treatment (*P* = 0.03). MeSA an aromatic HIPV produced via the shikimic acid pathway (Khan et al., 2020). Salicylic acid is known to coordinate plant defense responses to biotic stressors, such as pathogens, by promoting systemic acquired resistance (SAR) and increased expression of pathogen-related genes (Khan et al., 2020). In *Fusarium verticilliodes*-infected maize B73 silks, MeSA and auxin genes were upregulated, while jasmonic acid (JA) and ethylene (ET) genes were downregulated during induced systemic resistance (ISR) activity (Agostini et al., 2019). This indicates that B73 does possess the capacity for induced MeSA production, but it may be tissue dependent. Moreover, there may be tradeoffs between MeSA- and JA/ET-coordinated defense responses. For example, plant-growth promoting fungi *Trichoderma atroviridae* decreased maize landrace MeSA production by 78%, but increased JA in response to *Spodoptera frugiperda* herbivory, supporting the antagonistic relationship between MeSA and JA during herbivory-defense signaling (Contreras-Cornejo et al., 2018). However, we did not quantify JA in our study, so we are unable to conclude if the lack of MeSA corresponds to high JA concentrations in the stiff-stalk varieties. MeSA has been described interacting with parasitoid attraction. Snoeren et al. (2010) used MeSA knock-out *Arabidopsis thaliana* mutants, deficient in MeSA production to show that the mutant *A. thaliana* displayed increased attractiveness to the parasitoid wasp *Diadegma semiclausum* in a dose-dependent manner (Snoeren et al., 2010). Within maize, Yactayo-Chang et al. (2021) suggested that MeSA acts as a dose-dependent cue for host-selection by female *S. frugiperda*, as it significantly increased oviposition preference for B73 and B104 inbred maize genotypes (Yactayo-Chang et al., 2021). Alternatively, Salamanca et al., 2019 observed MeSA increased attractiveness of European corn borer (*Ostrinia nubilalis*) eggs to numerous predators, including adult lady beetles and predatory mites within cranberry bogs (Salamanca et al., 2019). It is clear that the beneficial presence or absence of MeSA is context dependent (Dicke and Baldwin, 2010), and is reliant an available reservoir of parasitoids and predators and interactions with antagonistic hormone signaling. However, the strong genotypic control of MeSA implies it may be an ideal target for breeding if the context is elucidated.

After MeSA, the top nine HIPV influenced were terpenoids: (e)-4,8-dimethyl-1,3,7-nonatriene, caryophyllene, trans-geranylgeraniol, linalool, β-myrcene, trans-α-bergamotene, epi-bicyclosesquiphellandrene, germacrene D, D-limonene. Terpenoids play a central role in plant-insect interactions and include a wide diversity of compounds (Block et al., 2019). Both non-volatile and volatile terpenoids play pivotal roles in responses to biotic stress. After MeSA, the top HIPVs ranked in this study were all members of the terpenoid family. Pests can physiologically respond to many terpenoids, determined by measured electroantennogram activity of *S. frugiperda* to maize headspace volatiles (Pinto-Zevallos et al., 2016). However, the response of insect pests to maize terpenoids may be genotype- and pest-specific (Yactayo-Chang et al., 2021). In fact, numerous studies support the genotype-specific nature of terpenoid synthesis (Gouinguené et al., 2001; Degen et al., 2004; Block et al., 2018). For example, Degen et al. (2004) showed strong variation in β-caryophyllene production when comparing American inbred and European cultivars (Degen et al., 2004), and it was later shown that American cultivars possessed low activity of *terpene synthase 23*, which produces β-caryophyllene, when compared to European Flint and teosinte varieties (Köllner et al., 2008). However, β-caryophyllene production increased in B73 and B104 post *S. frugiperda* feeding (Yactayo-Chang et al., 2021). Moreover, β-caryophyllene produced in maize roots attract entomopathogenic nematodes upon *Diabrotic virgifera virgifera* belowground herbivory. The production of β-caryophyllene in B73 is catalyzed by *terpene synthase 8*, due to the lack of expression of *terpene synthase 23* (Köllner et al., 2013). The impressive diversity and functional redundancy of terpene synthase enzymes ensures the genotype-specific, and tissue-dependent, nature of terpenoid release. However, this also highlights the necessity for future research regarding the context-dependent response of pest and pest-enemies to terpenoids, across maize genotypes (Michereff et al., 2019).

### Microbial Influence on Plant Growth Parameters

The rhizosphere microbiome was experimentally manipulated through sterilization and inoculation with plant growth promoting *Bacillus altitudinis*. While microbe treatments didn’t influence HIPV directly, the results of this study suggest an indirect mechanism of HIPV manipulation via changes in biomass allocation. However, the soil sterilization (BS, S) was the major driver of differences in both root and shoot biomass allocation (Figures 2, 3). Moreira et al. (2019) saw similar results when inoculating maize with *Ralstonia eutropha* and *Chryseobacterium humi* PGPR; *Ralstonia* and *Chryseobacterium* promoted root and shoot biomass under sterile conditions (Moreira et al., 2019). In our study, sterilization was used as a control for microbiome effects on plant growth and HIPV production. Yet, the only environment where a microbial inoculant would be added to sterile media is within built environments, such as indoor hydroponics cultivation (Trivedi et al., 2020). Built environments such as indoor agricultural systems are important for specific crops and conditions (Lawson et al., 2019) but using sterilized controls is not ideal, as most research exploring inoculant-driven biotic stress resiliency is aimed at field cultivation in industrial soil agroecosystems (Canfora et al., 2021). When paired with artificial conditions within the greenhouse context, patterns of plant-microbial interactions involved in tri-trophic signaling seen in HIPV elicitation may evade detection. Care should be taken during experimental planning to minimize artificial conditions which prevent accurate observation of ecological phenomena.

### When and Where Might Microorganisms Influence Herbivore-Induced Volatile Compounds and Herbivore Response?

There is mounting evidence of single strain *Bacillus* inoculants promoting plant growth and biotic stress resilience (Vardharajula et al., 2011; Huang et al., 2015; Sun et al., 2017; Saeid et al., 2018; de Lima et al., 2019; Hashem et al., 2019; Pranaw et al., 2020), but research is beginning to emphasize the importance of microbial consortia for PGP functions (Woo and Pepe, 2018; Hindersah et al., 2020). Consortia of PGPR involve two or more beneficial microorganisms, including bacteria and fungi, selected based on growth promoting traits such as nutrient provisioning, hormone production, antibiosis, etc. (Sekar et al., 2016). Trait stacking allows consortia to promote multi-stressor resiliency, amplifying the benefits of PGPR when compared to single-strain inoculums. Bradáčová et al. (2019) identified biomass yield responses of tomato (*Lycopersicum esculentum*) during growth in stressful (drought, P-deficiency, etc.) field conditions when inoculated with microbial consortia which included *Bacillus* species (Bradáčová et al., 2019). That study identified minimal benefits of single strain inoculants on tomato growth in field conditions. Interestingly, when single strain inoculants and consortia were compared in greenhouse cultivated tomatoes, the growth promoting effects were indistinguishable. The lack of PGP effect within the greenhouse was attributed to the absence of stress during greenhouse cultivation, whereby under these optimal conditions the host response to PGPR is determined solely by genetically-controlled mechanisms (Bradáčová et al., 2019). The environmental context for observable PGP traits echoes the importance of experimental design when studying tri-trophic interactions.

Microbiota exist within the remarkably complex soil matrix. Soil structure determines the physiochemical environment in microbial microhabitats, shifting the diversity and function of soil microbiomes. Agricultural manipulation of the biotic and abiotic components of the soil matrix, through crop species and management practices, imprint on soil leading to legacy effects, a term called plant-soil feedback (Pineda et al., 2017). A major limitation to the efficacy of inoculants, whether single strain or consortia, is persistence in rhizosphere of field crops, often because researchers on focus selecting microbial species based on growth promoting traits, ignoring rhizosphere competency requirements (Kaminsky et al., 2019). Manipulation of plant-soil feedbacks can overcome this challenge by enriching the soil matrix with the chemical and physical factors required for promoting microbial diversity and stimulating PGPR abundance (Blundell et al., 2020). Research has only just begun to explore plant-soil feedbacks and how they mediate insect herbivory (reviewed here by Pineda et al., 2017). A recent study with wheat observed greater total emissions of HIPV when grown in a soil inoculum originating from cover crop soil, as opposed to fallow soil (Malone et al., 2020), suggesting wheat grown in fallow rotation has a reduced capacity for recruiting pest predators. However, these effects are dependent on crop species and duration of agricultural management (Howard et al., 2020). In maize, herbivore-induced root exudation of benzoxazinoids recruited PGP *Pseudomonas putida* to the rhizosphere (Neal et al., 2012). But when the benzoxazinoids were metabolically processed by the soil microbiome, the byproduct caused a legacy effect on maize grown in subsequent plantings, conferring insect resiliency through modulating jasmonic acid signaling and defense priming (Hu et al., 2018) and reducing fall army worm (*Spodoptera frugiperda*) growth. Another study saw that maize grown in soil collected from polyculture, as opposed to monoculture, produced more volatile metabolites as well as benzoxazinoids, and had reduced herbivory from *Chilo partellus* larvae (Mutyambai et al., 2019). By inoculating potting mix with 10% live soil in our study, we disconnected the soil microorganisms from their native habitat, potentially altering the mechanisms by which they establish in the rhizosphere and interact with plant root systems for defense metabolite production. Therefore, identifying mechanisms controlling species-specific insect resistance through plant-soil, and plant-insect-soil feedbacks, should be prioritized for feature research endeavors.

### Study Limitations

Besides the limitations regarding greenhouse exploration of tri-trophic ecological interactions, this study did not identify changes in herbivore survival, or predator/parasitoid attraction due to genotype and microbiome treatment. Plants have various lines of defense against herbivore generalists (Ali and Agrawal, 2012), such as leaf secondary metabolites, which may directly reduce herbivore growth and survival (Bezemer and Van Dam, 2005). For example, Badri et al. (2013) observed an influence of soil microbiomes on the leaf metabolome which reduced *Trichopulsia ni* leaf feeding behavior, which was hypothesized to be due to variations in amino acid content (Badri et al., 2013). As one of the primary growth-promoting functions of PGPR is nutrient acquisition (Richardson et al., 2009; Nath et al., 2018), assessing variations in tissue nutrient status is important for understanding the multitude of pathways involved in tri-trophic signaling. Moreover, as plant breeding manipulates regulatory elements controlling defense pathways and secondary metabolite synthesis (Maag et al., 2015), assessing breeding-manipulations in direct, and indirect, inducible defenses is of key importance (Swanson-Wagner et al., 2012; Chen et al., 2015; Rowen and Kaplan, 2016).

Additionally, HIPV target organisms are predators and parasitoids which rely on insect pests for survival (Hoballah et al., 2004; Clavijo McCormick et al., 2012). Disentangling which volatile cues recruit predators and parasitoids is fundamental for understanding which HIPV should be targets for anthropogenic manipulation through breeding or plant-soil feedbacks (Degen et al., 2012). Evidence suggests that the difference between basal VOCs and HIPV production upon herbivory is important for parasitoid recruitment (Sobhy et al., 2012). Our study did not disseminate differences in VOC production pre- and post-herbivory, so we cannot conclude if genotype and microbiome alter maize VOC elicitation response to *Heliocoverpa zea.*

## Conclusion

In conclusion, this study identified limited efficacy of the microbiome in influencing the composition and relative abundances of herbivore-induced volatile compounds (HIPV) in maize. Maize genotype was a strong driver of HIPV abundance and composition, suggesting the capacity to manipulate the maize volatilome through breeding practices. *Bacillus altitudinis* AP-283 influenced plant growth parameters, pointing to an indirect mechanism of volatilome manipulation. Tri-trophic signaling between herbivores-plant-microorganisms is a complex and highly dynamic process. Future research should ensure inclusion of the relevant environmental contexts if the goal is to explore the contribution of the rhizosphere microbiome to inducible plant defense metabolites.

## Data Availability Statement

The datasets presented in this study can be found in online repositories. The names of the repository/repositories and accession number(s) can be found in the article/Supplementary Material.

## Author Contributions

EN, SR, and AK conceived and designed the project, EN carried out herbivory trial and data collection. SR carried out greenhouse procedures, microbiome, and statistical analyses and wrote the manuscript. EN, SR, and AK provided significant editorial comments. All authors contributed to the article and approved the submitted version.

## Conflict of Interest

The authors declare that the research was conducted in the absence of any commercial or financial relationships that could be construed as a potential conflict of interest.

## Publisher’s Note

All claims expressed in this article are solely those of the authors and do not necessarily represent those of their affiliated organizations, or those of the publisher, the editors and the reviewers. Any product that may be evaluated in this article, or claim that may be made by its manufacturer, is not guaranteed or endorsed by the publisher.
