# Supplementary material for: Herbivory Protection via Volatile Organic Compounds Is Influenced by Maize Genotype, Not Bacillus altitudinis-Enriched Bacterial Communities
Source: Front Microbiol. 2022 May 2;13:826635. doi: 10.3389/fmicb.2022.826635 (PMC9108721; doi:10.3389/fmicb.2022.826635)
Supplement: Supplementary file 1 [file Data_Sheet_1.docx]

**Herbivory protection via volatile organic compounds is influenced by maize genotype, not *Bacillus altitudinis*-enriched bacterial communities**

Sierra S. Raglin^1^, Angela D. Kent^1^, Esther N. Ngumbi^2*^

^1^Microbial Ecology Laboratory,Department of Natural Resources and Environmental Sciences, University of Illinois at Urbana-Champaign, Urbana, IL, USA 61801

^2^Departments of Entomology, University of Illinois at Urbana-Champaign, Urbana, IL, USA 61801

*Corresponding author:

520 Morrill Hall

505 S. Goodwin Ave

Urbana, IL 61801

enn@illinois.edu

**Supplemental Information**

16S rRNA Amplicon Sequencing Results

*16S rRNA Amplicon Sequencing*

In this study, of the 3,958,705 sequences, 97.4 % (3,853,963) were assigned to 19,278 OTUs. After the removal of de-replicated sequences and chimeras (104,742, 2.6%), the final number of OTUs was 17,971. The total read count across samples was 39,182,026. Per sample, the average count/sample was 315,984.081 ± 93,177.314 standard deviation. The OTU table was rarefied to account for variations in sequencing depth across samples, with a rarefaction depth of 121,315 counts/sample. One sample was removed due to abnormally low counts/sample (7,426 reads). The final OTU table contained 17,650 OTUs and 14,921,745 total reads.

*Bacterial 16S rRNA Diversity*

Composition of the rhizosphere microbiome was assessed using Non-metric multidimensional scaling (NMDS) of the Bray-Curtis dissimilarity matrix paired with Permutational Multivariate Analysis of Variance (PERMANOVA). Block was used as the conditional variable in the PERMANOVA. Strong separation appeared between the BL/L and the BS/S treatments (Supplemental Figure 1), indicating sterility was a major driver of bacterial 16S rRNA community composition. PERMANOVA analyses confirmed that Microbe (R^2^ = 0.21485, P = 0.001) influenced bacterial beta diversity. There was no significant effect of genotype or genotype x microbe treatment on Bray-Curtis dissimilarity matrix.

The influence of maize genotype and microbe treatment on alpha diversity metrics (Observed, Chao1 richness, and Shannon Evenness) were assessed using linear mixed effects models with the lmer() functions from lme4 package (Figure 2). Levene’s Test for homogeneity of variances, and Shapiro Wilks test were used to assess if models violated assumptions. Model summary was used to identify specific genotypes and/or microbial treatments influencing model significance. Microbe treatment significantly influenced observed (F-value = 35.9131, P < 0.0001), Chao1 richness (F-Value = 33.6406, P < 0.0001), and Shannon evenness index (F-Value = 38.7560, P < 0.0001). These effects were predominantly drive by the BS and S treatments (P < 0.007 for all models). However, non-stiff stalk genotype OH43 significantly influenced Chao1 richness (P = 0.0039), Shannon evenness index (P = 0.01099), and observed richness (P = 0.003871). OH43 also significantly interacted with S treatment in all three models (P < 0.023 for all models), implying a genotype-specific interaction between maize and soil bacterial richness.

Supplemental Figure 1. Non-metric multidimensional scaling ordination of Bacteria 16S rRNA community, using a Bray-Curtis Dissimilarity Matrix. Points are colored based on Microbe treatment: Bacillus in Live soil (BL), Bacillus in Sterilized soil (BS), Live soil (L), and Sterilized soil (S).

Supplemental Figure 2. Alpha diversity values for genotype and microbe treatments based on 16S rRNA bacterial richness parameters. Observed richness, Chao1 richness index, and Shannon’s Evenness index are displayed.

Supplemental Figure 3. Relative abundance of bacterial phyla based on 16S rRNA V4 amplicon Illumina NovaSeq sequencing. Treatment (Genotype x Microbe) is displayed on the x-axis. OTUs were averaged within each treatment (Genotype x Microbe) and then transformed to relative abundance.
